# Paternal immune activation-induced alteration of 28S rRNA-derived small RNAs in sperm reprograms offspring phenotypes

**DOI:** 10.1093/pnasnexus/pgaf381

**Published:** 2026-01-13

**Authors:** Chenxuan Li, Chenxi Liu, Meiling Tan, Jiangxue Cai, Lu Lu, Yiran Sun, Bin He

**Affiliations:** Key Laboratory of Animal Physiology and Biochemistry, Ministry of Agriculture and Rural Affairs, College of Veterinary Medicine, Nanjing Agricultural University, NO.1 Weigang, Nanjing City, Jiangsu Province 210095, PR China; Key Laboratory of Animal Physiology and Biochemistry, Ministry of Agriculture and Rural Affairs, College of Veterinary Medicine, Nanjing Agricultural University, NO.1 Weigang, Nanjing City, Jiangsu Province 210095, PR China; Key Laboratory of Animal Physiology and Biochemistry, Ministry of Agriculture and Rural Affairs, College of Veterinary Medicine, Nanjing Agricultural University, NO.1 Weigang, Nanjing City, Jiangsu Province 210095, PR China; Key Laboratory of Animal Physiology and Biochemistry, Ministry of Agriculture and Rural Affairs, College of Veterinary Medicine, Nanjing Agricultural University, NO.1 Weigang, Nanjing City, Jiangsu Province 210095, PR China; Key Laboratory of Animal Physiology and Biochemistry, Ministry of Agriculture and Rural Affairs, College of Veterinary Medicine, Nanjing Agricultural University, NO.1 Weigang, Nanjing City, Jiangsu Province 210095, PR China; Key Laboratory of Animal Physiology and Biochemistry, Ministry of Agriculture and Rural Affairs, College of Veterinary Medicine, Nanjing Agricultural University, NO.1 Weigang, Nanjing City, Jiangsu Province 210095, PR China; Key Laboratory of Animal Physiology and Biochemistry, Ministry of Agriculture and Rural Affairs, College of Veterinary Medicine, Nanjing Agricultural University, NO.1 Weigang, Nanjing City, Jiangsu Province 210095, PR China; MOE Joint International Research Laboratory of Animal Health & Food Safety, Nanjing Agricultural University, NO.1 Weigang, Nanjing City, Jiangsu Province 210095, PR China

**Keywords:** sperm, 28S-rsRNAs, paternal immune activation, intergenerational effect, behavior

## Abstract

Parental environmental exposures can induce transgenerational effects through epigenetic modifications in germ cells. Although paternal immune activation is implicated in transgenerational metabolic and neuropsychiatric disorders, the germline-encoded molecular vectors mediating this inheritance remain poorly understood. Here, we demonstrated that lipopolysaccharide-induced immune activation dynamically upregulated the abundance of 28S ribosomal RNA-derived small RNAs (28S-rsRNAs) in mouse sperm in a time-dependent manner. Furthermore, epididymal sperm maturation exhibited heightened susceptibility to acute immune perturbations compared with spermatogenic processes, and 28S-rsRNAs were selectively incorporated during their transit through the caput epididymis. Strikingly, zygotic microinjection of synthetic 28S-rsRNAs recapitulated paternal immune activation phenotypes, resulting in offspring exhibiting metabolic syndrome-like phenotypes, including obesity and impaired insulin sensitivity. Concurrently, these manipulated offspring displayed neurobehavioral abnormalities characterized by heightened anxiety-like and aggressive behaviors, accompanied by hippocampal transcriptomic alterations. Taken together, our findings demonstrate that sperm 28S-rsRNAs contribute to paternal immune activation-mediated programming of offspring behavioral and metabolic phenotypes and provide mechanistic insights into environment-germline interactions.

Significance Statement Our study centers on sperm-borne 28S-rsRNAs—a recently identified class of abundant small non-coding RNAs in sperm with poorly defined biological roles. We investigated whether paternal immune activation, a common environmental stressor, alters 28S-rsRNAs profiles to modulate offspring phenotypes. By integrating LPS-induced acute inflammation models, zygotic microinjection of synthetic 28S-rsRNAs, and transcriptomic analyses, we uncover a role for 28S-rsRNAs in regulating intergenerational metabolic and behavioral traits. These findings establish a foundation for developing diagnostic and therapeutic strategies to mitigate the adverse effects of paternal immune activation and other environmental stressors on offspring health.

## Introduction

The Developmental Origins of Health and Disease (DOHaD) theory has emerged as a pivotal framework in developmental biology. Evidence from mammalian models has demonstrated that environmental stresses, including diet, mental stress, inflammation, and toxin exposure, during the window of developmental reprogramming, particularly gametogenesis and embryonic development, can directly elevate the risk of adverse health outcomes in the offspring (1–4). While maternal environmental perturbations during gestation and their epigenetic reprogramming effects on offspring phenotypes have been well characterized (3, 5), emerging evidence substantiates that paternal exposures constitute critical mediators of intergenerational inheritance. Among these paternal stressors, paternal immune activation has garnered attention due to its capacity to disrupt diverse physiological features in offspring (6). Paternal preconceptional exposure to parasitic, bacterial, or viral infections has been demonstrated to alter offspring's social and anxiety-like behaviors in murine models (7–10). Our prior studies revealed metabolic abnormalities, including obesity and glucose intolerance, in offspring of paternal mice subjected to lipopolysaccharide (LPS)-induced inflammatory challenge (2). These phenotypic perturbations coincide with marked epigenetic alterations in sperm, providing mechanistic support for their role in mediating intergenerational transmission of paternal immune activation. Elucidating these mechanisms will not only refine the DOHaD theoretical architecture but also provide a scientific foundation for designing cross-generational health interventions.

Recent studies employing zygotic microinjection of total sperm RNA extracts or specific subsets of sperm small noncoding RNAs (sncRNAs), such as microRNAs (miRNAs) and tRNA-derived small RNAs (tsRNAs), have established causal evidence that the “sperm RNA code” mediates the transmission of environmental stresses from fathers to offspring (4, 11–19 ). Using a novel small RNA sequencing method, PANDORA-seq (panoramic RNA display by overcoming RNA modification aborted sequencing), it was shown that a specific class of small RNAs, derived from 28S rRNA and designated as 28S rRNA-derived small RNAs (28S-rsRNAs), is particularly abundant in sperm (20, 21). Interestingly, 28S-rsRNAs demonstrate dynamic involvement in stress adaptation, showing acute-phase-specific upregulation in murine inflammatory models (21). Moreover, these sncRNAs orchestrate embryonic proteostasis by fine-tuning translation machinery and mitochondrial oxidative phosphorylation (20), unveiling their dual role as stress sensors and developmental regulators. However, it remains unclear whether 28S-rsRNAs are involved in offspring phenotypic changes induced by paternal immune activation.

This study addresses 2 questions regarding sperm-borne 28S-rsRNAs: first, whether these small RNAs demonstrate responsiveness to paternal immune activation, and second, whether they functionally modulate phenotypes in offspring. Our findings reveal that paternal immune activation triggered substantial alterations in the profiles of 28S-rsRNAs within mature sperm during their transit through the caput epididymis. Functional analysis identified sperm 28S-rsRNAs as epigenetic mediators that transmit paternal immune activation signals, leading to metabolic and neurobehavioral disorders in offspring, thus solidifying their functional significance within the “sperm RNA code.”

## Results

### Acute immune activation temporally alters sperm 28S-rsRNAs profiles

**Fig. 1.**
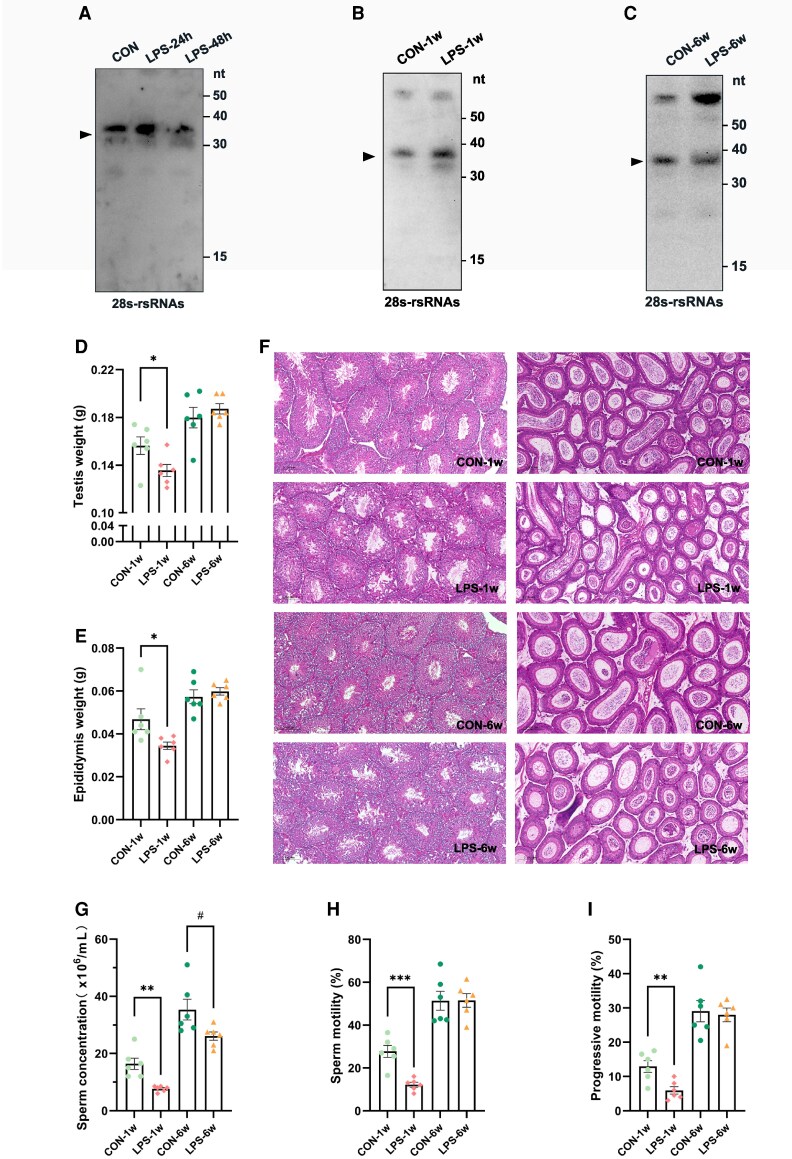
LPS-induced alterations in sperm 28S-rsRNAs abundance, reproductive organ weights, and sperm quality in male mice. A–C) The abundance of the sperm-borne 28S-rsRNAs of male mice with LPS treatment for 24/48 h (CON vs LPS-24 h vs LPS-48 h) A), 1 week (CON-1W vs LPS-1W) B), and 6 weeks (CON-6W vs LPS-6W) C). D) Testicular weights at 1 or 6 weeks post-LPS treatment. E) Epididymal weights at 1 or 6 weeks post-LPS treatment. F) Representative H&E staining of testis and epididymis at 1 or 6 weeks post-LPS treatment. G-I) Sperm concentration G), motility H), progressive motility I) at 1 or 6 weeks post-LPS treatment. Data are presented as mean ± SEM, n = 6. * indicates a significant difference (CON-1w vs LPS-1w, * means *P* < 0.05, ** means *P* < 0.01, *** means *P* < 0.001, **** means *P* < 0.0001), # indicates a significant difference (CON-6w vs LPS-6w, # means *P* < 0.05).

Existing evidence demonstrates that the epigenetic landscape of sperm remains environmentally sensitive throughout both spermatogenesis in the seminiferous tubules (approximately 35 days) and maturation in the epididymis (approximately 7 days) (22). No discernible changes were observed in the abundance of 30–40 nt 28S-rsRNAs in caudal epididymis sperm during the initial stages (24 or 48 h) of LPS treatment (Fig. 1A). However, an upregulation in the abundance of 28S-rsRNAs was noted 1 week after LPS treatment (Fig. 1B). Subsequently, following a 6-week recovery period, which encompasses an entire spermatogenic cycle, the alterations in 28S-rsRNAs abundance in sperm appeared to diminish (Fig. 1C). This temporal pattern indicates that paternal immune activation upregulates the abundance of 28S-rsRNAs within mature sperm during their transit through the caput epididymis.

Observations were conducted at various postinjection intervals to evaluate the systemic impact of acute immune activation on spermatogenesis and maturation. Male mice exhibited significant reductions in testicular (Fig. 1D) and epididymal weights (Fig. 1E) 1 week postinjection compared with control cohorts. Concurrently, LPS treatment for 1 week significantly decreased sperm count in the caput epididymis, with parallel declines in sperm concentration and motility observed in the cauda region (Fig. 1F–I). These pathological manifestations partially reversed after a 6-week recovery phase (Fig. 1F). Collectively, the data demonstrate transient physiological perturbations 1 week postintervention, whereas substantial recovery was observed by 6 weeks. Epididymal sperm maturation exhibits heightened susceptibility to acute immune activation compared with spermatogenic processes.

### Accumulation of 28S-rsRNAs in sperm during epididymal passage following acute immune activation

**Fig. 2.**
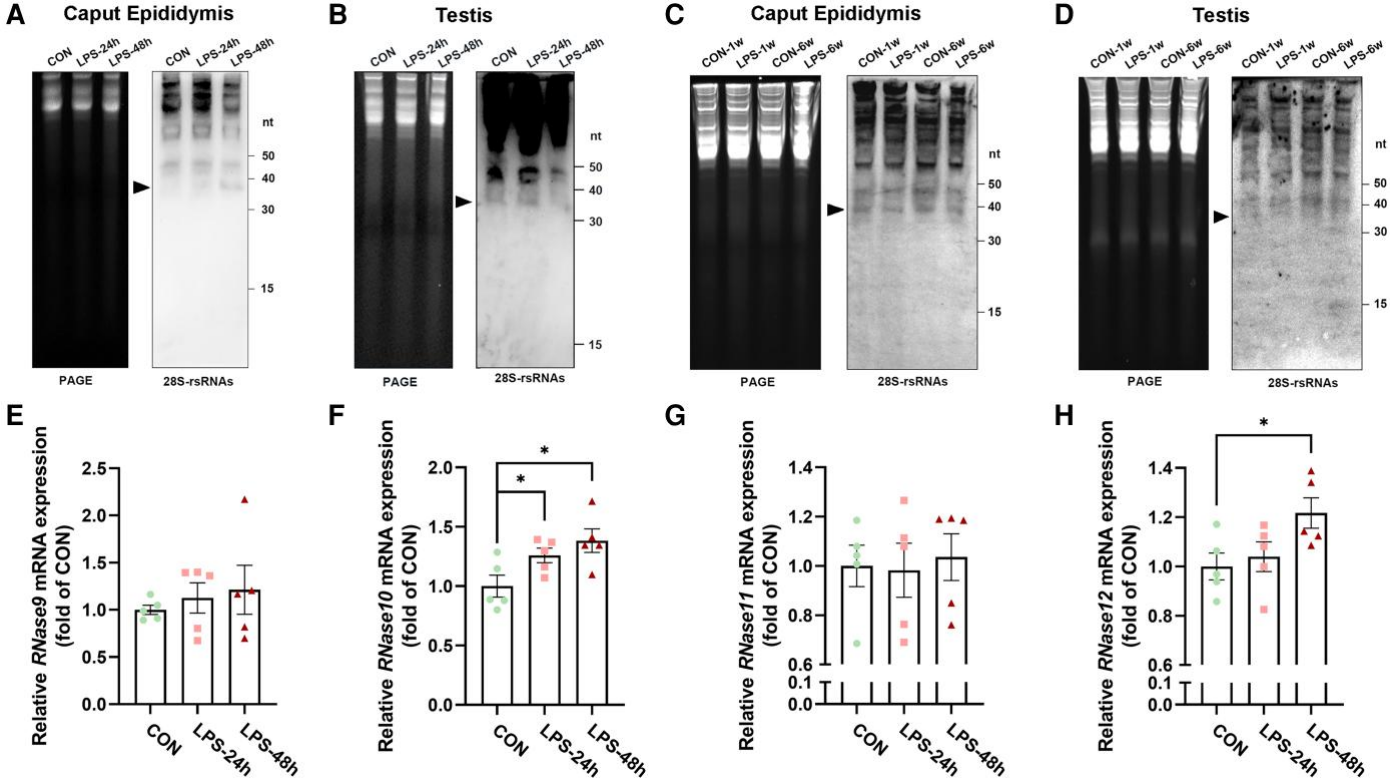
LPS-induced alterations in epididymal and testicular 28S-rsRNAs abundance and RNase A family gene expression levels in male mice. A and B) The expression of 28S-rsRNAs in the caput epididymis A) and testis B) after LPS treatment for 24 or 48 h. C and D) The expression of 28S-rsRNAs in the caput epididymis C) and testis D) after LPS treatment for 1 week or 6 weeks. Arrowheads denote 30–40 nt 28S-rsRNAs. E-H) Relative expression levels (fold changes vs CON) of epididymal RNase A family genes at 24 h/48 h post-LPS treatment of male mice: E) *RNase 9* expression levels; F) *RNase 10* expression levels; G) *RNase 11* expression levels; H) *RNase 12* expression levels. Data are presented as mean ± SEM, n = 5. * indicates a significant difference (vs CON, * means *P* < 0.05).

To explore the potential source of increased 28S-rsRNAs levels in sperm, we analyzed their expression in the caput epididymis and testis at various time points following LPS treatment. As expected, the acute inflammatory response was confirmed by a significant upregulation in the expression of inflammatory factors in both the testis and caput epididymis (Supplemental Fig. S1). We then investigated the dynamics of 28S-rsRNAs in these tissues following LPS challenge. Strikingly, the abundance of 28S-rsRNAs in the caput epididymis increased after 24 h of LPS treatment and became more pronounced at 48 h (Fig. 2A). In contrast, no comparable changes were detected in the testis at either time point (Fig. 2B), suggesting that the caput epididymis is the primary site responsive to acute immune activation for generating 28S-rsRNAs. Furthermore, the 28S-rsRNAs levels in both tissues returned to baseline by 1 week post-treatment and remained unchanged at 6 weeks (Fig. 2C and D). Given that sperm transit through the caput epididymis takes approximately 1 week, the concurrent timing of the transient increase in sperm 28S-rsRNAs abundance 1 week after LPS administration (Fig. 1B) strongly suggests that sperm are more likely to acquire these RNAs during their maturation in the caput epididymis, rather than in the testis. Further evidence supporting this hypothesis comes from the significant upregulation of the epididymis-specific ribonuclease (RNase) A family gene (23), *RNase 10*, at 24 h post-LPS treatment (Fig. 2E–H). Additionally, both *RNase 10* and *RNase 12* demonstrated significant increases in expression at 48 h post-LPS treatment (Fig. 2F, H), providing further credence to the speculation regarding the potential origin of 28S-rsRNAs.

### Zygotic microinjection of 28S-rsRNAs triggers altered growth and metabolic disorders in offspring

**Fig. 3.**
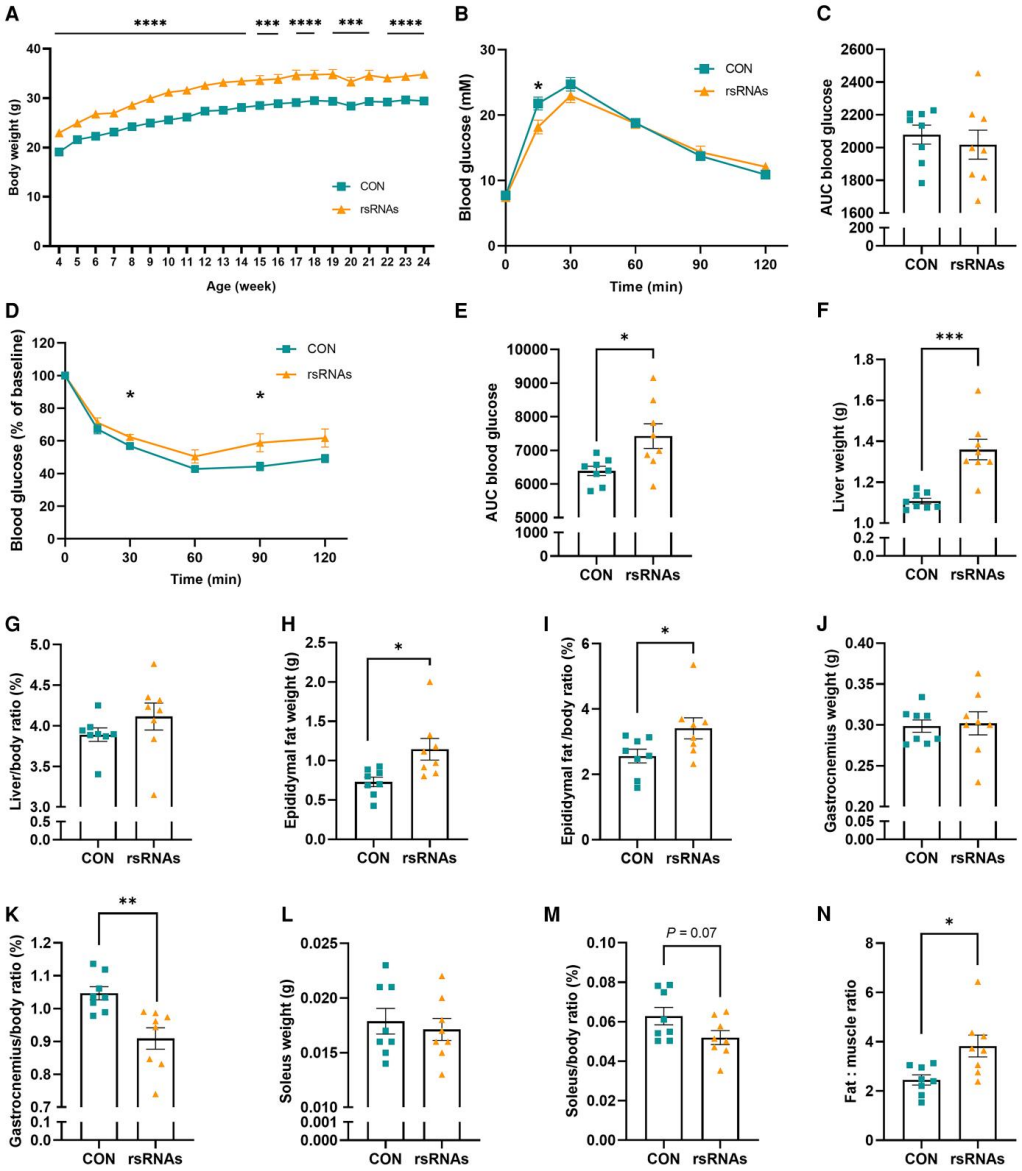
Zygotic microinjection of 28S-rsRNAs induces alterations in growth and glucose metabolism of male offspring. A) Growth dynamics of male offspring from scrambled RNA-microinjected (CON) or 28S-rsRNAs-microinjected (rsRNAs) zygotes. B) Blood glucose levels as assayed by GTT of F1 male mice at 18 weeks of age. C) AUC statistics for B). D) Blood glucose levels as assayed by ITT of F1 male mice at 19 weeks of age. E) AUC statistics for D). F-M) Organ weight and ratio (% organ/body weight) of male offspring: liver weights F) and ratios G); epididymal fat weights H) and ratios I); gastrocnemius weights J) and ratios K); soleus weights L) and ratios M); N) Fat-to-muscle ratio. Data are presented as mean ± SEM, n = 8. * indicates a significant difference (vs CON, * means *P* < 0.05, ** means *P* < 0.01, *** means *P* < 0.001, **** means *P* < 0.0001).

To gain further insight into the function of sperm 28S-rsRNAs and the potential consequences of their elevated abundance, a combinatorial pool of 28S-rsRNAs was synthesized and microinjected into zygotes using established sperm RNA transfer methodologies (2, 4). Compared with control F1 males, F1 males microinjected with 28S-rsRNAs exhibited significantly higher body weight throughout their growth cycle (Fig. 3A). Besides, the 28S-rsRNAs-microinjected offspring exhibited a subtle but consistently greater absolute body weight gain than controls from sexual maturity (around 8 weeks of age) onward (Supplemental Fig. S2). Although zygotic microinjection of 28S-rsRNAs did not significantly affect glucose tolerance in male offspring as assessed by glucose tolerance tests (GTT) (Fig. 3B and C), it significantly impaired insulin sensitivity as assessed by insulin tolerance tests (ITT) (Fig. 3D and E). An increase in liver weight (Fig. 3F and G) and epididymal fat mass (Fig. 3H and I) was accompanied by a reduction in muscle (gastrocnemius and soleus) proportions (Fig. 3J–M), leading to a markedly elevated fat-to-muscle ratio (Fig. 3N). These results demonstrate that microinjection of 28S-rsRNAs resulted in the development of obesity and metabolic syndrome-like phenotypes in male offspring.

### Zygotic microinjection of 28S-rsRNAs induces anxiety-like and aggressive behaviors in offspring

**Fig. 4.**
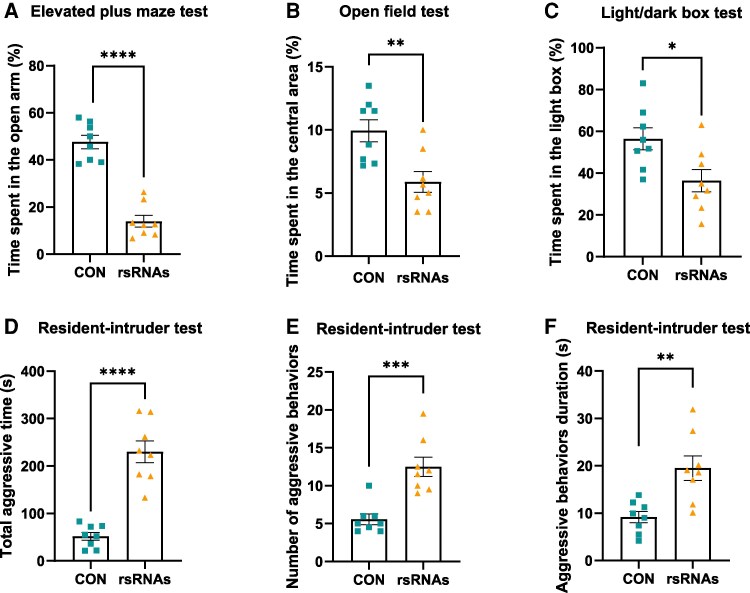
Zygotic microinjection of 28S-rsRNAs induces behavioral alterations in male offspring. A–C) Anxiety-related behavior test of male offspring from scrambled RNA-microinjected (CON) or 28S-rsRNAs-microinjected (rsRNAs) zygotes. Percentage of time in the open arm (% total time) during the elevated plus maze test A). Percentage of time in the central area (% total time) during the open field test B). Percentage of time in the light box (% total time) in the light/dark box test C). D-F) Resident—intruder test of male offspring. Total duration of aggressive behaviors (% total time) D). Number of occurrences of aggressive behavior E). Mean duration per aggressive behavior F). Data are presented as mean ± SEM, n = 8. * indicates a significant difference (vs CON, * means *P* < 0.05, ** means *P* < 0.01, *** means *P* < 0.001, **** means *P* < 0.0001).

Since paternal immune activation disrupts the nervous system of offspring, we conducted behavioral tests on the male offspring to determine if alterations in 28S-rsRNAs could produce similar behavioral modulation. The male offspring mice in the 28S-rsRNAs-microinjection group exhibited anxiety-like behaviors, as evidenced by their significantly shorter durations in the open arm of the elevated plus-maze test (Fig. 4A), the central area of the open field test (Fig. 4B), and the light box of the light/dark box test (Fig. 4C). Furthermore, the 28S-rsRNAs mice displayed markedly increased levels of aggressive behaviors (Fig. 4D–F). These results suggest that zygotic microinjection of 28S-rsRNAs, mimicking an increase in their abundance in sperm, leads to anxiety-like and aggressive behaviors in the offspring.

### Zygotic microinjection of 28S-rsRNAs alters hippocampal gene expression in offspring

**Fig. 5.**
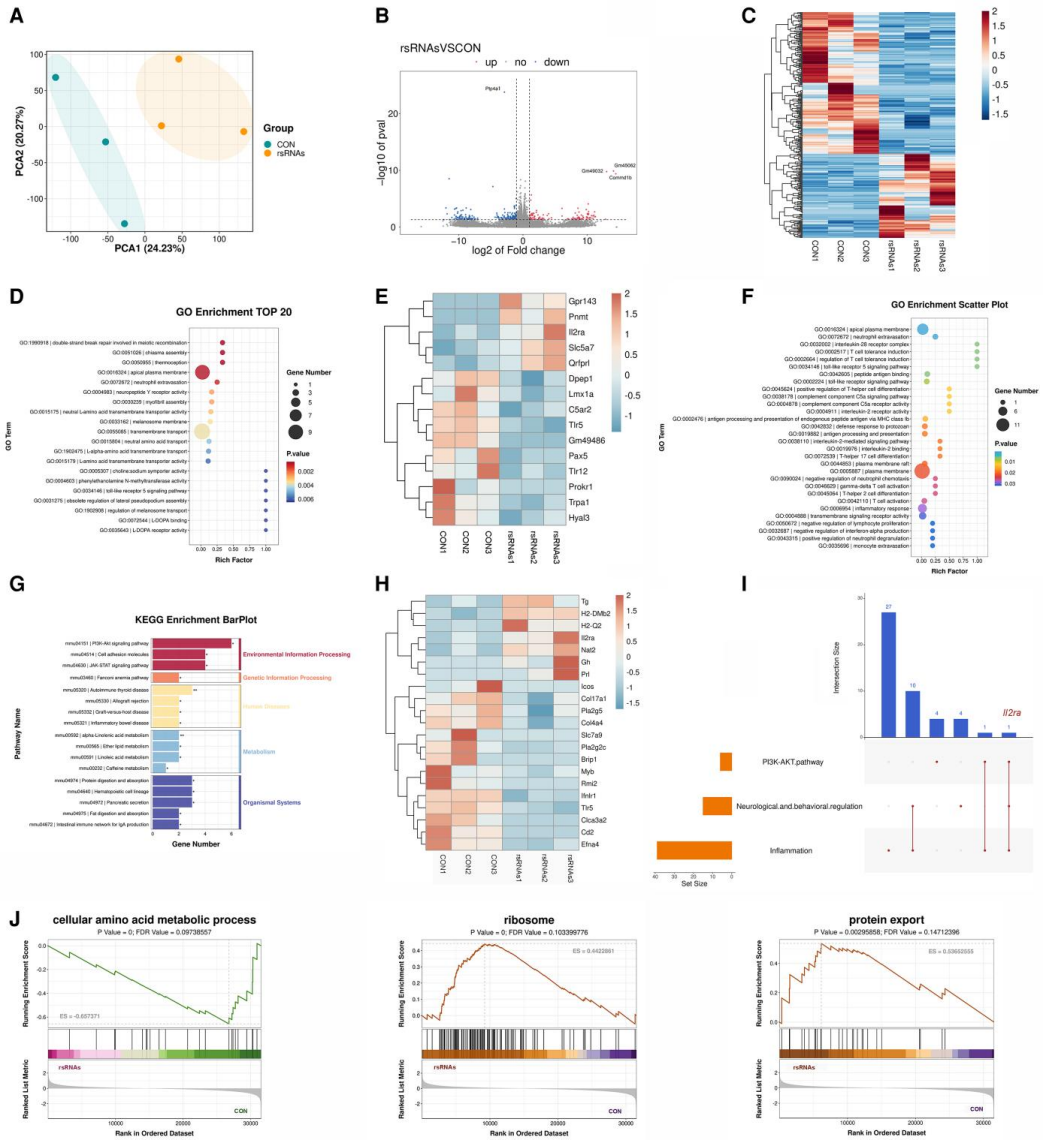
Zygotic microinjection of 28S-rsRNAs induces alterations in hippocampal gene expression in male offspring. A–C) Transcriptomic profiling of the hippocampus in male offspring derived from zygotes microinjected with scrambled RNA (CON) or 28S-rsRNA (rsRNAs). PCA illustrating sample distribution A). Volcano plot of differentially expressed genes (DEGs, z-score normalized expression) B). Heatmap of all DEGs C). D–F) GO enrichment analysis of DEGs. Top 20 enriched GO terms (ranked by *P* values) (D). Heat map showing the expression of neurological and behavioral-associated genes (z-score normalized expression) E). Top 30 enriched GO terms associated with immune functions (ranked by *P* values) F). G and H) KEGG pathway enrichment analysis of DEGs. Significantly enriched KEGG pathways (* indicates a significant difference vs CON, * means *P* < 0.05, ** means *P* < 0.01, **** means *P* < 0.001, **** means *P* < 0.0001) G). Heat map showing the expression of KEGG analysis-associated genes (z-score normalized expression) H). I) UpSet plot showing the interactions between DEGs enriched for inflammatory processes, neuro-/behavioral regulation (GO), and the PI3K/AKT signaling pathway (KEGG). J) Gene Set Enrichment Analysis (GSEA): left, enrichment analysis of selected GO term; middle/right, enrichment analysis of selected KEGG pathways.

To better understand the mechanisms involved, we examined the transcriptional profiles of the hippocampus, a key brain region for behavioral regulation. Principal component analysis (PCA) of the hippocampal transcriptomes revealed a distinct segregation of the 28S-rsRNAs-microinjected and control offspring (Fig. 5A). A total of 320 Differentially expressed genes (DEGs) were identified in the male F1 offspring of the 28S-rsRNAs group, including 119 upregulated and 201 downregulated genes (Fig. 5B and C). Among these, protein tyrosine phosphatase 4a1 (*Ptp4a1)* gene was identified as significantly downregulated, while the most prominently altered upregulated gene cluster included *Gm49032*, *Gm45062,* and copper metabolism domain containing 1b gene (*Commd1b*) (Fig. 5B). Gene ontology (GO) analysis revealed 185 significantly enriched terms in biological processes, 26 in cellular components, and 63 in molecular functions (Fig. 5D). Consistent with the observed behavioral phenotypes, GO analysis demonstrated significant enrichment of terms related to neurological and behavioral regulation, such as neuropeptide Y receptor activity, L-DOPA receptor activity, L-DOPA binding, phenylethanolamine N-methyltransferase activity, and choline: sodium symporter activity (Fig. 5D). Expression analysis of the 15 associated DEGs further confirmed their distinct regulation across groups (Fig. 5E). Additionally, significant enrichment was detected for GO terms encompassing innate and adaptive immunity, inflammatory responses, and related signal transduction pathways (Fig. 5F). Kyoto Encyclopedia of Genes and Genomes (KEGG) pathway analysis identified 17 significantly enriched pathways involving 21 DEGs (Fig. 5G and H). Among these, the PI3K-AKT signaling pathway, which is known to be implicated in neuroimmune crosstalk, contained the highest number of DEGs (Fig. 5G). Furthermore, the interleukin 2 receptor subunit alpha (*Il2ra*) gene was located at the intersection of this pathway and the gene sets for inflammatory processes and neuro/behavioral regulation (Fig. 5I), suggesting its potential role as a key regulatory molecule. Additionally, Gene Set Enrichment Analysis (GSEA) showed that genes associated with the “cellular amino acid metabolic process” were downregulated in the hippocampus of the injected group offspring, while pathways related to “ribosome” and “protein export” were activated (Fig. 5J). Together, these results demonstrate that microinjection of 28S-rsRNAs alters hippocampal gene expression networks involved in neurotransmitter release, immune signaling, and protein synthesis.

## Discussion

While paternal immune activation triggered by diverse pathogens (parasitic, bacterial, and viral) has been recognized to reprogram offspring neurodevelopmental trajectories and metabolic homeostasis (7–9), the germline-encoded molecular drivers of this phenomenon remain poorly characterized. Our study addresses this knowledge gap by revealing that LPS-induced paternal immune activation specifically remodels the sperm 28S-rsRNA profiles. To investigate the functional consequences of this change, we modeled its effect by performing zygotic microinjection of synthetic 28S-rsRNAs. Offspring mice subjected to zygotic microinjection of 28S-rsRNAs exhibited altered growth and metabolic disorders. Notably, the 28S-rsRNAs-microinjected offspring demonstrated elevated aggression and anxiety-like behaviors, correlating with dysregulation of hippocampal gene expression. These results identify sperm-borne 28S-rsRNAs as a epigenetic vector mediating the intergenerational effects of paternal immune activation.

Recently, it was confirmed that rsRNAs, especially 28S-rsRNAs, have the highest abundance in mouse sperm (20). Elucidating the biogenesis of sperm sncRNAs is critical, as it establishes a mechanistic conduit linking paternal environmental exposures to intergenerational phenotypic outcomes in offspring. It has been shown that 28S-rsRNAs are actively involved in the acute phase of inflammation (21), but their function is unclear. The source of sperm-borne sncRNAs, particularly where they are synthesized and incorporated into sperm during their transition from the testis (24, 25) to the epididymis (13, 26), has been debated. In the present study, during the acute phase of immune activation in mice (24- and 48-h postinduction), a significant increase in 28S-rsRNAs abundance was observed in the caput epididymis, whereas no comparable changes were detected in the testis. Consistently, the epididymis-specific genes of the RNase A family involved in small RNA processing were up-regulated in the caput epididymis (23). Further investigation revealed an increase in the abundance of 28S-rsRNAs in sperm during the transition period from the caput to the cauda epididymis (about 1 week) following LPS treatment. When the recovery period covering the entire cycle of spermatogenesis up to the arrival of mature sperm in the cauda epididymis (about 6 weeks) (27), no observable alteration in the abundance of 28S-rsRNAs within the sperm was observed. These results strongly suggest that sperm acquired 28S-rsRNAs during their passage through the caput epididymis.

Most evidences suggest that epididymal epithelial cells deliver sncRNAs to sperm via extracellular vesicles during caput epididymal transit (13, 26, 28). Given that sperm contain rRNAs and tRNAs, these molecules can be processed into rsRNAs and tsRNAs under stress conditions. Our previous findings have shown that oxidative stress reshapes the mature sperm sncRNA profile, providing support for this phenomenon (29). Furthermore, since sperm express LPS receptors, toll-like receptor 2/4 (TLR2/TLR4), which are capable of responding to LPS (30, 31), we propose that during epididymal transit, they are directly exposed to LPS or other inflammatory mediators present in the luminal fluid. Such exposure could thereby trigger the intracellular generation of specific RNA fragments, such as 28S-rsRNAs, within sperm. Therefore, paternal immune activation-induced alteration of 28S-rsRNAs in sperm may result from a combination of active incorporation of epididymis-derived fragments and in situ processing of endogenous RNA precursors within sperm. This dual-origin model, while supported by our data and existing literature, warrants further direct experimental investigation.

The regulatory role of sperm 28S-rsRNAs in offspring physiological traits was further validated. In this study, zygotic microinjection of a synthesized 28S-rsRNAs pool recapitulated paternal immune activation phenotypes, resulting in offspring exhibiting metabolic disorders, including obesity and impaired insulin sensitivity. It is well known that sperm 30–40 nt sncRNAs (predominantly tsRNAs and rsRNAs) mediate intergenerational transmission of paternally acquired metabolic disorders (4, 11). Our present results indicate that the microinjection of rsRNAs alone, in the absence of tsRNAs, can also induce metabolic disorders in offspring. Concurrently, these manipulated offspring display neurobehavioral abnormalities characterized by heightened anxiety-like and aggressive behaviors. Previous studies demonstrate that paternal immune activation induced by LPS or polyinosinic: polycytidylic acid reprograms sperm sncRNA profiles-specifically PIWI-interacting RNA and miRNA expression, thereby inducing anxiety and depressive disorders across generations (7–9). Our results provide evidence that sperm 28S-rsRNAs could serve as a conduit for transmitting environmental stresses from fathers to offspring.

Sperm-borne sncRNAs, including miRNAs, tsRNAs, and the more recently identified rsRNAs, have been implicated in mediating the intergenerational effects of paternal environmental exposures (4, 11–19). Our previous work in a paternal immune activation model supports a specific role for angiogenin-mediated tsRNAs in this process (2). Although the present study identifies 28S-rsRNAs as a functionally relevant vector, they are unlikely to act alone. Instead, we propose that a “sperm RNA code”—comprising a consortium of multiple small RNA species, collectively orchestrates the transmission of paternal environmental signals. The findings indicate that 28S-rsRNAs are key components within this complex network, thereby expanding the repertoire of epigenetically active molecules in sperm. Furthermore, post-transcriptional modifications add a critical layer of regulatory complexity by influencing the stability and function of these sncRNAs (4). This is consistent with our observation that zygotic microinjection of synthetic 28S-rsRNAs, which lacked native-like modifications, only partially recapitulated the offspring metabolic disturbances following paternal inflammation (2). This discrepancy suggests that the mechanistic actions of 28S-rsRNAs may involve both modification-dependent and modification-independent pathways. Thus, while our results establish 28S-rsRNAs as functional vectors of paternal influence, the absence of physiological modifications in our synthetic constructs represents a key experimental limitation. Future studies should dissect the functional contributions of distinct sncRNA classes and define how specific RNA modifications influence their stability, targeting, and regulatory capacity in intergenerational inheritance.

The hippocampal transcriptome is known to mediate behavioral abnormalities and susceptibility to paternal environmental insults. Consistent with this, microinjection of 28S-rsRNAs led to altered expression of inflammatory genes in the offspring hippocampus, suggesting the establishment of a persistent, low-grade immunostressed microenvironment. GO analysis further highlighted alterations in key regulators of anxiety-related neurotransmission, including neuropeptide Y and L-DOPA receptor activities, as well as phenylethanolamine N-methyltransferase activity (32–35). These findings support a model in which hippocampal inflammation links paternal immune experience to offspring behavior through disruption of these neurotransmitter systems (36, 37). KEGG pathway analysis identified the PI3K-Akt pathway as the most significantly enriched, functioning as a signaling hub that integrates both immune and neurotransmitter cues (37, 38). Notably, hippocampal *Il2ra* upregulation emerged as a pivotal link between paternal immune stress and offspring neurobehavioral deficits. We hypothesize that *Il2ra* activation aggravates neuroinflammation and disrupts the delicate balance of synaptic plasticity and neurotransmitter signaling, potentially through the PI3K-AKT pathway and other downstream effectors (39). This convergence of immune and neural dysregulation ultimately provides a plausible molecular substrate for the observed hippocampal dysfunction and anxiety-like behavior. In addition, GSEA revealed pronounced enrichment of protein translation-related processes, marked by downregulation of intracellular amino acid metabolism and upregulation of ribosome biogenesis and protein export pathways. Given the established role of these pathways in translational control (40), the findings collectively point to a widespread dysregulation of protein synthesis machinery in the hippocampus. Taken together, these findings demonstrate that zygotic microinjection of 28S-rsRNAs selectively remodels hippocampal gene expression networks involved in neurotransmitter signaling, immune activation, and protein synthesis.

The mechanism by which zygotic 28S-rsRNAs overexpression leads to hippocampal reprogramming is complex and indirect, potentially mediated by reprogramming early embryonic development, which cascades into altered neurodevelopment. This concept is supported by studies showing that 28S-rsRNAs can drive lineage differentiation in murine embryoid bodies and embryonic stem cells (20). Furthermore, Pan et al. (41) provided a plausible mechanistic link by showing that rsRNAs can directly target metabolic genes via an RNAi-like mechanism, thereby regulating embryonic and placental development. While the detailed pathway from the zygote to the adult hippocampus remains to be fully mapped, these findings suggest that 28S-rsRNAs act by reprogramming fundamental developmental and metabolic processes, initiating a cascade that culminates in altered hippocampal gene expression and function.

It is also important to acknowledge the limitations of our current study. First, our functional investigations, including the metabolic, behavioral, and transcriptomic analyses, were conducted exclusively in male offspring. Whether female offspring exhibit similar or distinct phenotypic and molecular changes in response to paternal immune activation or zygotic 28S-rsRNAs microinjection remains an open and critical question for future research. Second, as previously mentioned, the synthetic 28S-rsRNAs used in our microinjection experiments lack the post-transcriptional modifications present in their endogenous counterparts, which may account for the partial recapitulation of the paternal immune activation phenotype. Future studies employing sex-balanced designs and incorporating physiologically modified RNAs will be essential to fully elucidate the scope and mechanisms of 28S-rsRNA-mediated intergenerational inheritance.

Taken together, our data showed the origin of sperm 28S-rsRNAs and their role in regulating offspring phenotypes: (i) sperm acquired 28S-rsRNAs during their passage through the caput epididymis and (ii) 28S-rsRNAs are crucial for paternal immune activation-mediated programming of offspring behavioral and metabolic phenotypes.

## Materials and methods

### Animals

Male C57BL/6J mice were housed under specific pathogen-free (SPF) conditions at Nanjing Agricultural University's Laboratory Animal Center. All experimental procedures were performed following the Institutional Animal Care and Use Committee (IACUC)-approved guidelines of Nanjing Agricultural University (NJAU No. 20230419063).

To establish the immune activation model, 7-week-old male C57BL/6J mice were administered a single intraperitoneal (i.p.) injection of LPS (5 mg/kg body weight) derived from Escherichia coli O111: B4 (L2630, Sigma-Aldrich). Control animals received equivalent volumes of sterile saline. LPS solutions were prepared in endotoxin-free water containing 0.9% (w/v) NaCl. Tissue samples from both experimental and control cohorts were collected at predetermined intervals (24, 48 h, 1 week, or 6 weeks postinjection).

### Sperm isolation and quality assessment

Mature sperm samples were isolated from the mouse cauda epididymides using established protocols (2). Bilateral cauda epididymides were carefully trimmed to remove adipose and other tissue from male mice and then incubated in 5 ml of preheated phosphate-buffered saline (PBS, 37°C). Four to six incisions were made in each cauda epididymis, followed by a 15-min incubation to facilitate sperm release. Collect an appropriate volume of the sample for sperm quality assessment. Sperm were evaluated using the mobile computer-assisted sperm analyzer iSperm® mCASA (Aidmics Biotechnology) (42), following the instructions for use of the software provided by the manufacturer. At least 4 fields of each sample were captured at 30 frames per second. The sperm suspension was filtered through a 40-μm mesh to remove tissue debris and treated on ice with somatic cell lysis buffer (40 min) to eliminate somatic cell contamination. Following centrifugation (600 × *g*, 5 min, 4°C), pellets underwent 2 cycles of ice-cold PBS washing via sequential resuspension and centrifugation. Purified sperm were homogenized in TRIzol reagent for RNA isolation.

### Northern blots

Northern blotting was performed using established protocols (2, 20). RNA was extracted from caudal epididymal sperm (pooled from 3 mice), the caput epididymis, or the testis, and separated on 15% urea-polyacrylamide gels. Target RNA was visualized via UV imaging after NA-Red staining (1:2000 dilution; D0128, Beyotime). The electrophoresed RNA was transferred to a positively charged nylon membrane (FFN13, Beyotime) and fixed by cross-linking at 80°C for 2 h. Membranes were prehybridized in ULTRAhyb® buffer (AM8670, Invitrogen) at 42°C (≥1 h), followed by overnight hybridization (12–16 h) with 16 nM Digoxigenin (DIG)-labeled probes (Supplemental Table S1; GENEWIZ-synthesized) at 42°C. Posthybridization processing included sequential washing cycles at 42°C: two 15-min low-stringency washes (2× SSC, 0.1% SDS) and two 5-min high-stringency washes (0.1× SSC, 0.1% SDS), followed by 10-min rinsing in 1× SSC. Blocking was performed using Roche blocking buffer (11096176001) for 2 h at room temperature, after which membranes were incubated with anti-DIG-AP Fab fragments (1:10,000 dilution in fresh blocking buffer; 11093274910, Roche) for 30 min. Membranes underwent four 15-min washes in maleic acid buffer (1×, 0.3% Tween-20), equilibrated in detection buffer (0.1 M Tris-HCl, 0.1 M NaCl, pH 9.5) for 5 min and incubated with CSPD substrate (Roche, 11755633001) at 37°C (15 min, dark). Chemiluminescent signals were acquired using a VersaDoc 4000MP system (Bio-Rad).

### Histological analysis

For histomorphological evaluation, testes and caput epididymides were dehydrated, embedded in paraffin, and sectioned into 5-μm slices. The hematoxylin and eosin (H&E) staining was performed on tissue sections following established histological protocols, followed by microscopically analyzing (BX63F OLYMPUS Micro Image System, OLYMPUS).

### Quantitative RT-PCR

Total RNA was extracted using TRIzol reagent (15596026, Invitrogen) following the manufacturer's instructions. Quantitative RT-PCR (RT-qPCR) was performed as previously described (2). Following RNA isolation, reverse transcription of 1 μg total RNA was performed using *TransScript*® Uni All-in-One First-Strand cDNA Synthesis SuperMix (AU341-02, TransGen Biotech Co., Ltd). RT-qPCR was performed on a QuantStudio 6 Flex Real-Time PCR System (Thermo Fisher Scientific) with *PerfectStart*® Uni RT & qPCR Kit (AUQ-01, TransGen Biotech Co., Ltd) in 10 μL reaction volumes, according to the manufacturer's protocols. Gene expression quantification was performed using the 2^−ΔΔCt^ method, with primer sequences (Tsingke Biotech; Supplemental Table S1) designed for target-specific amplification.

### Oocyte collection, zygote microinjection of synthetic 28S-rsRNAs, and embryo transfer

Embryo collection and transfer were performed following established protocols (2). In vitro fertilization (IVF) assessment using metaphase II oocytes (identified by first polar body extrusion) demonstrated successful fertilization through 2-pronuclear formation. Synthetic 28S-rsRNAs or scrambled RNAs (Supplemental Table S1) were diluted to 2 ng/μL and delivered into zygotic male pronuclei via Leica-assisted microinjection following established methodologies (4, 11). Resultant zygotes were surgically implanted into pseudopregnant ICR strain surrogate dams.

### Blood glucose examination during GTT and ITT

Glucose metabolism tests were performed in 18- to 19-week-old mice. For GTT and ITT, mice were fasted for 12 and 6 h, respectively, followed by intraperitoneal injection of glucose (2 g/kg body weight) or insulin (0.75 IU/kg body weight, 12584-58-6, Aladdin). Blood glucose levels of tail vein blood were assayed at 0 (baseline), 15-, 30-, 60-, and 120-min postinjection using a glucometer (ACCU-CHEK Active Blood Glucose Meter, Roche) (2). Areas under the curve (AUC) for blood glucose during GTT and ITT were calculated using the trapezoidal rule.

### Behavioral tests

#### Open field test

The open field test was performed in a 40 × 40 × 30 cm opaque arena under standardized illumination. The central area was defined as a 20 × 20 cm area in the middle (43). Mice were individually placed in the center and allowed to freely explore for 10 min. The whole procedure was videotaped, and the time spent by each mouse in the central area was analyzed.

#### Light/dark box test

The light/dark box apparatus consisted of 2 adjacent compartments: a darkened chamber (one-third of the arena) and an illuminated chamber (two-thirds), separated by an opaque partition with a central aperture. Mice were positioned in the darkened chamber at test initiation, and exploratory behavior was video-recorded for 5 min. The proportion of total time occupied in the light zone was then quantified.

#### Elevated plus maze test

The elevated plus maze comprised 2 open arms (40 × 9.5 cm) and 2 enclosed arms (40 × 9.5 × 9.5 cm), separated by a central platform (9.5 × 9.5 cm) elevated to 40 cm (44). At test onset, mice were positioned in the central zone. Exploratory behavior was monitored during a 5-min session, with time spent in open versus enclosed arms quantified.

#### Resident–intruder paradigm

The test mice were housed in isolation for a period exceeding seven days, during which the bedding remained unchanged. The test began with the introduction of an unfamiliar male mouse, slightly younger and smaller in size, as an intruder into the home cage, and video recordings were made for 5 min (45). The analysis focused on the total duration of aggressive behaviors, the frequency of aggressive acts, and the average duration of each aggressive episode. The experimental protocols and behavioral evaluation criteria were adapted from established methodologies described in published literature (46–48 ).

### RNA-seq

Total RNA was isolated from the hippocampus of male offspring mice using TRIzol-based extraction and purification. The RNA libraries were constructed and then sequenced on the Illumina NovaseqTM 6000 platform by LC Bio-Technology CO., Ltd (Hangzhou, China) following standard procedure. The raw sequencing reads from all samples were aligned to the *Mus musculus* reference genome (mm10) using the HISAT2 package (version: hisat2-2.2.1). The mapped reads for each sample were then assembled into transcripts using StringTie (version: stringtie-2.1.6) with its default parameters. Subsequently, transcriptomes from all samples were merged to reconstruct a comprehensive transcriptome using the gffcompare software (version: gffcompare-0.9.8). Following the generation of the final transcriptome, StringTie and ballgown were employed to estimate the expression levels of all transcripts. Expression abundance for mRNAs was quantified using the FPKM (Fragments Per Kilobase of transcript per Million mapped reads) metric. Differential gene expression analysis between comparative groups was conducted using the DESeq2 software. DEGs were identified using thresholds of | log_2_(fold change) | ≥1 and *P* < 0.05. GO and KEGG pathway enrichment analysis were performed on significant DEGs (*P* < 0.05). GSEA was implemented via GSEA software (v4.1.0) with MSigDB gene sets to detect coordinated expression changes in predefined GO terms and KEGG pathways. Gene sets meeting |normalized enrichment score (NES)| >1, nominal (NOM) *P* < 0.05, and false discovery rate q < 0.25 were classified as statistically significant. Expression patterns of significantly enriched DEGs were visualized via z-score normalized values using OmicStudio (LC Bio-Technology CO., Ltd, Hangzhou, China). PCA was performed utilizing the princomp function available in the R statistical computing environment.

### Statistical analysis

All data are presented as mean ± standard error of the mean (SEM) and were analyzed using the independent samples *t* test and One-way ANOVA using SPSS 26.0 software (IBM Corp., USA). Statistical significance was defined as *P* < 0.05.

## Supplementary Material

pgaf381_Supplementary_Data

## Data Availability

Data supporting the findings of this manuscript are available in the main text and in the Supplementary material.
