# Supplementary material for: Paternal immune activation-induced alteration of 28S rRNA-derived small RNAs in sperm reprograms offspring phenotypes
Source: PNAS Nexus. 2026 Jan 13;5(1):pgaf381. doi: 10.1093/pnasnexus/pgaf381 (PMC12797211; doi:10.1093/pnasnexus/pgaf381)
Supplement: pgaf381_Supplementary_Data [file pgaf381_supplementary_data.pdf]

PNAS nexus

Supplementary Information for

Paternal immune activation-induced alteration of 28S rRNA-derived small RNAs in sperm reprograms offspring phenotypes

Chenxuan Li[1], Chenxi Liu[1], Meiling Tan[1], Jiangxue Cai[1], Lu Lu[1], Yiran Sun[1], Bin He[1, 2*]

[1] Key Laboratory of Animal Physiology & Biochemistry, Ministry of Agriculture and Rural Affairs, College of Veterinary Medicine, Nanjing Agricultural University, Nanjing, 210095, PR China;

[2] MOE Joint International Research Laboratory of Animal Health & Food Safety, Nanjing Agricultural University, Nanjing 210095, PR China.

*To whom correspondence should be addressed: **Email:** heb@njau.edu.cn

This PDF file includes:

Figures S1 to S2
Tables S1

Fig. S1. Expression of inflammatory cytokines in the caput epididymis and testis with or without LPS treatment for 24 and 48 h

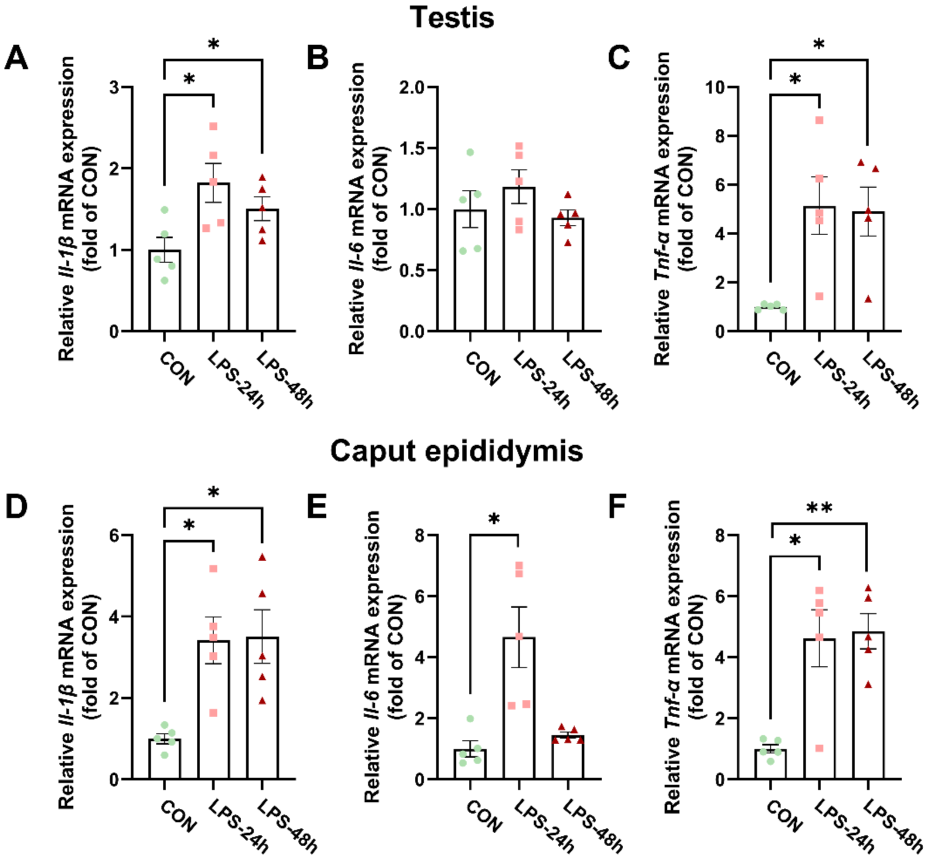

A-C Relative expression levels (fold changes vs. CON) of testis inflammatory factors genes at 24 h/48 h post-LPS treatment of male mice: *Il-1β* expression levels (A); *Il-6* expression levels (B); *Tnf-α* expression levels (C). D-F Relative expression levels (fold changes vs. CON) of epididymal inflammatory factors genes at 24 h/48 h post-LPS treatment of male mice: *Il-1β* expression levels (D); *Il-6* expression levels (E); *Tnf-α* expression levels (F). Data are presented as mean ± SEM, n = 5. ∗ indicates a significant difference (vs. CON; ∗ means $P < 0.05$; ∗∗ means $P < 0.01$).

Fig. S2. Post-weaning body weight gain in male offspring

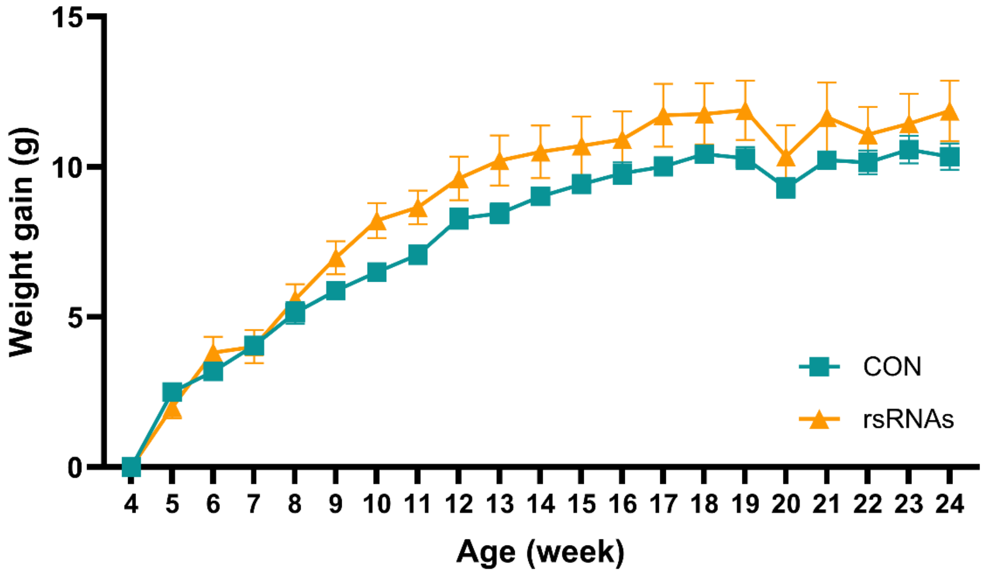

Weekly Body weight gain was normalized to the weight at weaning (4 weeks of age) for each animal. Data are presented as mean ± SEM.

Table S1. Primers, synthetic RNA, and probe sequences used in this study

Primers for qRT-PCR	Sequence (5' to 3')
RNase9-F	CCCGTAGAGGAGCACTTTTG
RNase9-R	GCACAGGAGGAGGAAGCTAAA
RNase10-F	AAAAGCCCTCAGCACACAAG
RNase10-R	GTCACCTTCATTTTGCCTACACT
RNase11-F	AGACCTGCCTTGAGACAAGATCA
RNase11-R	TGTTTGGATATGCTGAGGCCA
RNase12-F	CCCCTGATGGTCCTAATGGTG
RNase12-R	CTGCATCTGTGGTCGGGTTC
Il-1β-F	CTTCAGGCAGGCAGTATC
Il-1β-R	CAGCAGGTTATCATCATCATC
Il-6-F	GCCTTCTTGGGACTGATGCT
Il-6-R	TGACAGGTCTGTTGGGAGTGG
Tnf-α-F	GACGTGGAACTGGCAGAAGA
Tnf-α-R	ACTGATGAGAGGGAGGCCAT
β-actin-F	GTACCACCATGTACCCAGGC
β-actin-R	AACGCAGCTCAGTAACAGTCC
**Synthetic RNAs for microinjection**	**Sequence (5' to 3')**
rsRNAs-28s	P-CGCGACCUCAGAUCAGACGUGGCGACCCGCUGAAU<br>P-CGCGACCUCAGAUCAGACGUGGCGACCCGCUGAAUUUAAGC
Scrambled RNA	CCUCCCAAAGUGCUGGGAUUACAGGCGUGAG
**Probe for Northern blot**	**Sequence (5' to 3')**
rsRNAs-28s	DIG-CGGGTCGCCACGTCTGATCTGAGGTCGCG
